# A Structure–Activity Relationship Study on the Antioxidant Properties of Dithiocarbamic Flavanones

**DOI:** 10.3390/antiox13080963

**Published:** 2024-08-08

**Authors:** Mihail Lucian Birsa, Laura Gabriela Sarbu

**Affiliations:** Department of Chemistry, Alexandru Ioan Cuza University of Iasi, No. 11 Carol I Boulevard, 700506 Iasi, Romania

**Keywords:** flavanones, flavonoids, antioxidants, benzopyrans, dithiocarbamates, radical enolates

## Abstract

The antioxidant properties of 3-dithiocarbamic flavanones have been investigated. The influence of the halogen substituents on ring A of the flavanones and the nature of the secondary amine from the dithiocarbamic moiety have been accounted. The results indicated that the presence of a halogen substituent at the C-8 position of the benzopyran ring induce better antioxidant properties against DPPH and ABTS than butylated hydroxytoluene (BHT) and ascorbic acid. The presence of a halogen substituent at the mentioned position appears to induce a higher stability for a free radical intermediate at the C-3 position of the benzopyran ring. A free radical enolate is most likely to be involved in the antioxidant activity of this dithiocarbamic flavanone. It is a stable intermediate that supports the influence of dithiocarbamic moiety on the antioxidant properties of the reported flavanones.

## 1. Introduction

Reactive oxygen species (ROS) are unstable derivatives of molecular oxygen that display high reactivity. ROS are divided into two main categories, namely radical ROS, such as superoxide (O_2_^•−^), peroxyl (HO^•^) or hydroxyl radicals (ROO^•^), and non-radical ROS, such as singlet oxygen (^1^O_2_) and hydrogen peroxide (H_2_O_2_). In humans, ROS are generated mainly by mytochondrial processes, as well as by various enzymes during normal cell metabolism [1,2]. At low concentrations, ROS can act as mediators in a number of signaling processes; however, at higher concentrations, ROS indiscriminately damage biomacromolecules such as DNA, proteins, lipids and carbohydrates [3,4]. In the human body, this kind of oxidative stress can be counteracted to some degree with the help of antioxidants. These, in turn, can be produced by the cells (endogenous) and include enzymes, non-enzymatic proteins, thiols, etc., or acquired from the diet (exogenous), such as vitamins, carotenoids, polyphenols, etc. [5].

Flavonoids are a group of polyphenols found in nature throughout the plant kingdom. In plants, they play a wide variety of roles, acting as antioxidants, modulators, pollinator attractors or pest-deterrents [6,7,8,9,10,11]. Flavonoids inevitably make their way into our diets and it is estimated that the dietary intake of flavonoids is up to 800 mg/day [5]. As such, considerable effort has been dedicated to study the effects of flavonoids on the human body. Flavonoids have been shown to display cardioprotective, neuroprotective, antiproliferative, antiviral and antimicrobial properties [12,13,14,15,16,17,18,19]. In the last few years, our group reported on the synthesis, antimicrobial and cytotoxic properties of several novel synthetic flavonoids bearing a 1,3-dithiolium ring fused to the C ring of the flavan core [20,21]. Herein, we report on the antioxidant properties of several synthetic dithiocarbamic flavanones, used as precursors in the synthesis of the above mentioned tricyclic synthetic flavonoids.

## 2. Materials and Methods

### 2.1. Chemistry

Melting points were obtained using a KSPI melting point meter (A. KRÜSS Optronic, Hamburg, Germany) and are uncorrected. IR spectra were recorded on a Bruker Tensor 27 instrument (Bruker Optik GmbH, Ettlingen, Germany). NMR spectra were recorded on a Bruker 500 MHz spectrometer (Bruker BioSpin, Rheinstetten, Germany). Chemical shifts are reported in ppm downfield from TMS. Mass spectra were recorded on a Thermo Scientific ISQ LT instrument (Thermo Fisher Scientific Inc., Waltham, MA, USA). UV-vis measurements were carried out using a Varian BioChem 100 spectrophotometer (Varian, Sydney, Australia) equipped with a 8 × 6 multicell block water thermostatted. All reagents were commercially available and used without further purification. Elemental analysis and copies of ^13^C-NMR spectra are available in the Supplementary Material.

#### 2.1.1. General Procedure for 6,8-Dibromo-2-(4-chlorophenyl)-4-oxochroman-3-yl-pyrrolidine-1-carbodithioate (**5h**)

To a solution of 1-(3,5-dibromo-2-hydroxyphenyl)-1-oxoethan-2-yl-pyrrolidine-1-carbodithioate (**3h**) (0.44 g, 1 mmol) in a mixture of MeOH/CHCl_3_ (15 mL, 1:1 *v*/*v*), aminal **4** (0.3 g, 1 mmol) was added and the reaction mixture was heated under reflux for 3 h. After cooling, the solid material was filtered off and purified by recrystallization from ethanol to give **5h** (0.45 g, 80%) as colorless crystals. M.p. 184–185 °C. IR (ATR, cm^−1^) 2893, 1700, 1586, 1431, 1088, 1431, 1088, 800, 640, 539, 458. ^1^H NMR (CDCl_3_, selected data for the major *syn* isomer) δ 8.03 (d, *J* = 2.1 Hz, 1H), 7.90 (d, *J* = 2.1 Hz, 1H), 7.47 (d, *J* = 8.3 Hz, 2H), 7.37 (d, *J* = 8.3 Hz, 2H), 6.26 (d, *J* = 3.5 Hz, 1H), 5.99 (d, *J* = 3.5 Hz, 1H), 3.91 (m, 1H), 3.87 (m, 1H), 3.66 (m, 1H), 3.52 (m, 1H), 2.03 (m, 2H), 1.97 (m, 2H). ^13^C NMR (CDCl_3_, selected data for the major *syn* isomer) δ 187.4, 186.4, 155.5, 141.5, 134.7, 133.3, 129.5, 128.8, 128.1, 122.2, 114.8, 113.2, 80.8, 56.7, 56.6, 50.8, 26.0, 24.2. MS (EI) *m*/*z*: 558.95 (M^+^, 3%) for C_20_H_16_^79^Br_2_ClNO_2_S_2_.

#### 2.1.2. 6,8-Dibromo-2-(4-chlorophenyl)-4-oxochroman-3-yl-piperidine-1-carbodithioate (**5i**)

M.p. 170–171 °C, (0.45 g, 79%). IR (ATR, cm^−1^) 2945, 1693, 1585, 1452, 1268, 813, 653, 543. ^1^H NMR (CDCl_3_, selected data for the major *syn* isomer) δ 8.04 (d, *J* = 2.2 Hz, 1H), 7.98 (d, *J* = 2.2 Hz, 1H), 7.47 (d, *J* = 8.1 Hz, 2H), 7.37 (d, *J* = 8.1 Hz, 2H), 6.35 (d, *J* = 3.6 Hz, 1H), 6.00 (d, *J* = 3.6 Hz, 1H), 4.25 (m, 2H), 3.84 (m, 2H), 1.69 (m, 6H). ^13^C NMR (CDCl_3_, selected data for the major *syn* isomer) δ 190.6, 186.0, 155.8, 141.6, 135.0, 133.3, 129.5, 128.8, 128.7, 122.3, 114.6, 113.1, 80.9, 57.4, 54.2, 52.1, 26.1, 25.3, 24.1. MS (EI) *m*/*z*: 572.86 (M^+^, 1%) for C_21_H_18_^79^Br_2_ClNO_2_S_2_.

#### 2.1.3. 6,8-Dibromo-2-(4-chlorophenyl)-4-oxochroman-3-yl-morpholine-4-carbodithioate (**5j**)

M.p. 167–168 °C, (0.43 g, 75%). IR (ATR, cm^−1^) 2914, 1702, 1587, 1447, 1259, 1214, 808, 644, 540. ^1^H NMR (CDCl_3_, selected data for the major *syn* isomer) δ 8.04 (d, *J* = 2.3 Hz, 1H), 7.90 (d, *J* = 2.3 Hz, 1H), 7.45 (d, *J* = 8.4 Hz, 2H), 7.37 (d, *J* = 8.4 Hz, 2H), 6.29 (d, *J* = 3.7 Hz, 1H), 6.01 (d, *J* = 3.7 Hz, 1H), 4.20 (m, 2H), 3.86 (m, 2H), 3.73 (m, 4H). ^13^C NMR (CDCl_3_, selected data for the major *syn* isomer) δ 192.2, 186.1, 155.4, 141.6, 134.9, 133.1, 129.5, 128.8, 128.2, 122.2, 114.9, 113.2, 80.7, 66.2, 66.1, 57.1, 51.0, 50.7. MS (EI) *m*/*z*: 574.88 (M^+^, 3%) for C_20_H_16_^79^Br_2_ClNO_3_S_2_.

#### 2.1.4. 6,8-Diiodo-2-(4-chlorophenyl)-4-oxochroman-3-yl-pyrrolidine-1-carbodithioate (**5m**)

M.p. 205–206 °C (0.42 g, 75%). IR (ATR, cm^−1^) 2880, 1694, 1426, 1154, 952, 808, 639, 537. ^1^H NMR (DMSO-*d*_6_, selected data for the major *syn* isomer) δ 8.42 (d, *J* = 2.1 Hz, 1H), 8.04 (d, *J* = 2.1 Hz, 1H), 7.57 (d, *J* = 8.6 Hz, 2H), 7.51 (d, *J* = 8.6 Hz, 2H), 6.41 (d, *J* = 2.8 Hz, 1H), 5.89 (d, *J* = 2.8 Hz, 1H), 3.71 (m, 1H), 3.66 (m, 1H), 3.52 (m, 1H), 3.43 (m, 1H), 1.98 (m, 2H), 1.84 (m, 2H). ^13^C NMR (DMSO-*d*_6_, selected data for the major *syn* isomer) δ 187.5, 186.4, 158.9, 152.3, 135.8, 135.6, 134.7, 129.0, 128.8, 121.8, 89.8, 87.3, 80.1, 56.4, 56.3, 51.5, 26.1, 24.2. MS (EI) *m*/*z*: 654.34 (M^+^, 4%) for C_20_H_16_ClI_2_NO_2_S_2_.

#### 2.1.5. 6,8-Diiodo-2-(4-chlorophenyl)-4-oxochroman-3-yl-piperidine-1-carbodithioate (**5n**)

M.p. 195–196 °C (0.41 g, 72%). IR (ATR, cm^−1^) 2928, 1698, 1434, 1255, 963, 824, 623, 512, 458. ^1^H NMR (DMSO-*d*_6_, selected data for the major *syn* isomer) δ 8.40 (d, *J* = 1.4 Hz, 1H), 8.00 (d, *J* = 1.4 Hz, 1H), 7.52 (m, 4H), 6.41 (d, *J* = 2.5 Hz, 1H), 5.95 (d, *J* = 2.5 Hz, 1H), 4.10 (m, 2H), 3.77 (m, 2H), 1.50 (m, 6H). ^13^C NMR (DMSO-*d*_6_, selected data for the *syn* major isomer) δ 190.3, 186.2, 159.0, 152.4, 135.6, 135.5, 134.2, 130.2, 128.8, 121.9, 89.8, 86.8, 82.2, 57.1, 54.1, 52.2, 26.2, 25.5, 23.8. MS (EI) *m*/*z*: 668.94 (M^+^, 2%) for C_20_H_18_ClI_2_NO_2_S_2_.

### 2.2. In Vitro Antioxidant Activities

#### 2.2.1. 2,2-Diphenyl-1-picrylhydrazyl radical (DPPH) Assay

The DPPH method was performed according to the reported literature procedure [22,23]. Using a standard 4 mL quartz UV cell, flavanones **5a**–**o**, at different concentrations (1.6–2.5 mM in ethanol), were added to a solution of DPPH (0.1 mM in ethanol, 2.75 mL). The flavanones were added in 80 µL batches until absorbance dropped below half of the initial value. The reaction for scavenging DPPH radicals was performed in the dark at room temperature and the absorbance was measured at 517 nm after 15 min of incubation at 37 °C. The IC_50_ (nM) was determined as the flavanones’ **5a**–**o** concentration of 50% of the DPPH scavenging capacity (Table 1).

#### 2.2.2. 2,2′-Azino-bis(3-ethylbenzothiazoline-6-sulfonic acid) (ABTS) Assay

The ABTS assay was performed according to a slightly modified literature-reported protocol [24,25]. In short, ABTS radical cation (ABTS^+^) was prepared by reacting an ABTS solution (7 mM) in phosphate buffer (PBS, 0.1 M, pH 7.4) with a solution of potassium persulfate (2.45 mM) in water. The mixture was left to stand at room temperature for 16 h before use. After that, the mixture was diluted with PBS to reach an absorbance value of 0.70 ± 0.5 at 734 nm. Using a standard 4 mL quartz UV cell, flavanones **5a**–**o**, at different concentrations (0.8–2.5 mM in ethanol), were added to a solution of ABTS^+^ (2.75 mL). The flavanones were added in 20 µL batches until absorbance dropped below half of the initial value. The reaction for scavenging ABTS^+^ radicals was performed in the dark at room temperature and the absorbance was measured at 734 nm after 10 min of incubation at 37 °C. The IC_50_ (nM) was determined as the flavanones’ **5a**–**o** concentration of 50% of the ABTS^+^ scavenging capacity (Table 1).

#### 2.2.3. Ferric Reducing Antioxidant Power (FRAP) Assay

The FRAP method was performed according to the reported literature procedure. The FRAP reagent was prepared by mixing acetate buffer (300 mM, pH 3.6), a solution of 10 mM 2,4,6-tris(2-pyridyl)triazine (TPTZ) in 40 mM HCl, and 20 mM FeCl_3_ at 10:1:1 (*v*/*v*/*v*). Using a standard 4 mL quartz UV cell, flavanones **5a**–**o**, at different concentrations (1.8–2.4 mM in ethanol), were added to a solution of FRAP (2.75 mL). The flavanones were added in 30 µL batches until absorbance dropped below half of the initial value. The reaction for scavenging radicals was performed in the dark at room temperature and the absorbance was measured at 593 nm after 20 min of incubation at 37 °C. The IC50 (nM) was determined as the flavanones’ **5a**–**o** concentration of 50% of the FRAP scavenging capacity (Table 1).

## 3. Results

### 3.1. Chemistry

**Scheme 1:**
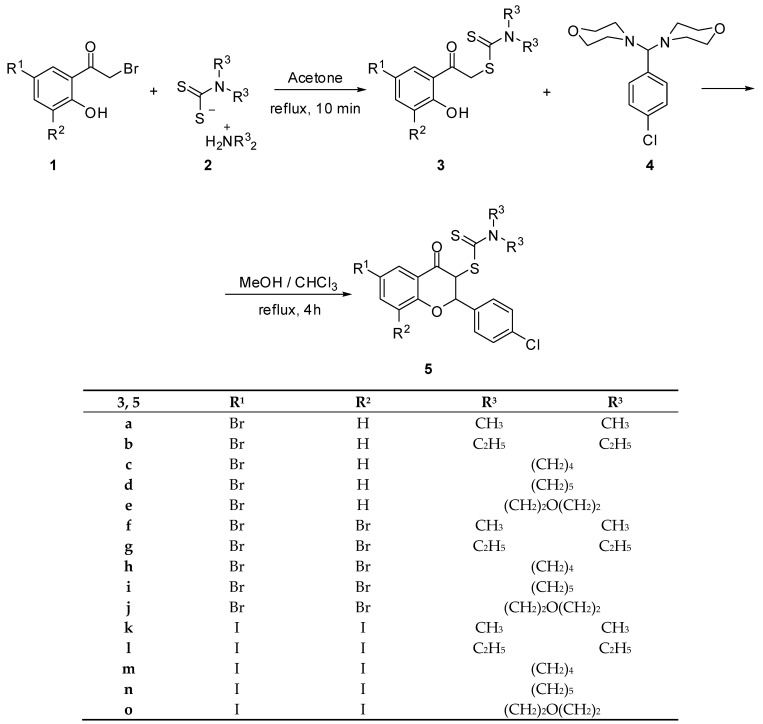
Synthesis of 3-dithiocarbamic flavanones **5a**–**o**.

The synthesis of 3-dithiocarbamic flavanones **5a**–**o** has been accomplished through a two-step procedure, as shown in Scheme 1: the synthesis of the corresponding phenacyl carbodithioates **3**, followed by their cyclocondensation to the corresponding substituted benzopyran derivatives. The key precursors for phenacyl dithiocarbamates **3a**–**o** are 2-bromo-1-(5-bromo-2-hydroxyphenyl)ethan-1-one [26], 2-bromo-1-(3,5-dibromo-2-hydroxyphenyl)ethan-1-one [26] and 2-bromo-1-(2-hydroxy-3,5-diiodophenyl)ethan-1-one [27], compounds that have been synthesized according to the reported procedures. The reactions of these compounds with the various salts of dialkyldithiocarbamic acid **2**, which are easily available from the reaction of secondary amine with carbon disulfide, have provided the corresponding phenacyl dithiocarbamates **3a**–**o** in good yields (70–80%). The treatment of the latter compounds with *p*-chloroaminal **4** has been performed using a recently reported experimental procedure by us, providing the desired 3-dithiocarbamic flavanones **5a**–**o** (Scheme 1).

The synthesis of flavanones **5a**–**g**, **5k**,**l** [20] and **5o** has been previously reported by us. The structure of newly synthesized flavanones has been proved by spectral and analytical data. The formation of the benzopyran ring is accompanied by important spectral changes. ^1^H-NMR spectra indicated the disappearance of the phenolic proton signal, as well as of the methylene protons signal, and the appearance of two new doublets, corresponding to the hydrogen atoms from the C-2 and C-3 positions of the pyran ring. Besides these, the NMR pattern of the *para*-substituted aromatic ring originating from aminal **4** can be observed. ^13^C NMR spectra indicate the appearance of a new signal provided by the thiocarbonyl carbon atom around 190–192 ppm. The presence of two new signals, one for the C-2 carbon atom, found to be around 80.0 ppm, and one for the C-3 carbon atom, found to be around 60.0 ppm, also support the closure of the pyrane ring.

**Scheme 2:**
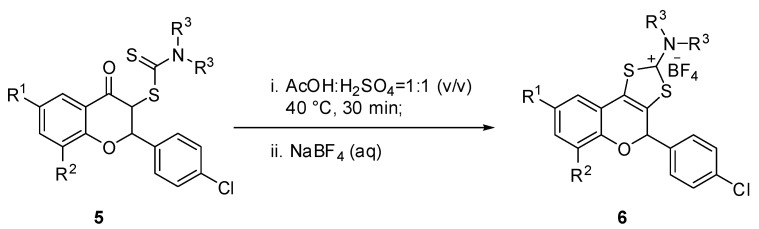
Synthesis of tricyclic flavonoids **6**.

As previously disclosed by us, the 3-dithiocarbamic flavanones are important precursors for a new class of tricyclic synthetic flavonoids of type **6**, which exhibit remarkable antimicrobial properties with MIC and MBC values of up to 0.24 µg/mL. The experimental procedure consists in acid catalyzed cyclocondensation of 3-dithiocarbamic flavanones, using a mixture of acetic acid/sulfuric acid (1:1 *v*/*v*) as cyclization agent, under mild conditions (Scheme 2). In order to investigate the antioxidant properties of tricyclic flavonoids, we synthesized a series of these compounds, namely **6a**–**g**, **6k**, **6l** and **6o**, whose synthesis and structural characterization have been previously reported by us.

### 3.2. In Vitro Antioxidant Activities

In order to investigate the antioxidant properties of the above synthesized compounds, we determined the DPPH, ABTS^+•^ and FRAP scavenging activities of these. Ascorbic acid and BHT, two common antioxidants, were used as standards for the antioxidant potential comparison [28,29,30]. The experimental procedure is described in Section 2.2; this was also used to determine the antioxidant properties of tricyclic flavonoids of type **6**. The results are presented in Table 1.

## 4. Discussion

In order to investigate the antioxidant properties of 3-dithiocarbamic flavanones of type **5**, we decided to undertake a structure–activity relationship study by varying different substituents at ring A and dithiocarbamic moiety, while keeping the same substituent (chlorine) at para position of ring B. There are three sets of dithiocarbamic flavanones, namely 6-bromo, 6,8-dibromo and 6,8-diiodo, each one with five derivatives according to the nature of secondary amine moiety, namely dimetylamino, diethylamino, pyrrolidinyl, piperidinyl and morpholinyl.

The analysis of the data presented in Table 1 disclosed interesting facts about structure–activity relationship of flavanones **5a**–**o**. Thus, the presence of a second halogen substituent at the C-8 position of the benzopyran ring of flavanones **5f**–**o** increased both DPPH and ABTS^+•^ scavenging activity as compared to the monohalogenated flavanones **5a**–**e**. In fact, the latter flavanones do not exhibit better antioxidant properties than both ascorbic acid and BHT. By introducing a second halogen substituent on the benzopyran ring, better antioxidant properties have been recorded. Thus, DPPH radical scavenging of flavanones **5f**–**j** and **5k**–**o** indicated better antioxidant activities as compared with BHT. All five 6,8-dibromoflavanones **5f**–**j** display smaller IC_50_ values than BHT, with pyrrolidinyl **5h** and *N*-morpholinyl **5j** derivatives being the most active with IC_50_ values of around 120 nM. From the 6,8-diiodoflavanones **5k**–**o** set, *N*-morpholinyl **5o** derivative displayed the best antioxidant properties with IC_50_ of 127 nM.

The ABTS^+•^ radical scavenging of flavanones **5f**–**j** and **5k**–**o** indicated better antioxidant activity as compared with both ascorbic acid and BHT. With the exception of **5i**, among the 6,8-dibromoflavanones **5f**–**j** set, all compounds displayed smaller IC_50_ values than the used standards, with special attention paid to the pyrrolidinyl derivative **5h** with an IC_50_ value of 10 nM. As compared with ascorbic acid, 6,8-diiodoflavanones **5l** and **5o** displayed better antioxidant properties. Again, *N*-morpholinyl derivative **5o**, with an IC_50_ of 13 nM, showed increased antioxidant activity as compared with both ascorbic acid and BHT.

**Scheme 3:**
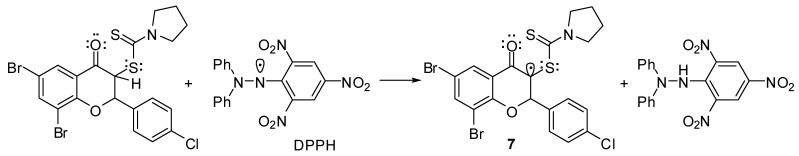
Plausible radical intermediate for flavanones’ radical scavenging mechanism.

Concerning the reaction mechanism for radical scavenging of flavanones **5**, important information have been provided by the investigation of antioxidant properties of tricyclic flavonoids of type **6**. DPPH, ABTS^+•^ and FRAP scavenging activities of tricyclic flavonoids revealed practically no antioxidant properties as compared with standards. Whereas the heterocyclocondensation of flavanones **5** to tricyclic flavonoids **6** (Scheme 2) is accompanied by the elimination of a hydrogen atom from the C-3 position of the benzopyran ring, it might be concluded that this atom makes the major contribution to the antioxidant properties of flavanones. Thus, a free hydrogen radical transfer to DPPH or ABTS^+•^ should provide a stable enolate radical of type **7** (e.g., Scheme 3).

The presence of the sulfur atom, with its non-participating electrons, next to the radical center also contributes to an increased stabilization due to a parallel overlapping with the free radical orbital. This intermediate also accounts for the influence of secondary amine moiety on radical scavenging of 3-dithiocarbamic flavanones, with *N*-pyrrolidinyl and *N*-morpholinyl carbodithioates being the most active. The formation of such a radical intermediate also explains the increased antioxidant activities of 6,8-disubstituted flavanones **5f**–**o** towards monosubstituted flavanones **5a**–**e**, apparently due to a supplementary stabilization induced by the C-8 bromine or iodine substituent. However, the formation of a free radical at the C-2 position, which is a benzylic one, cannot be ruled out.

## 5. Conclusions

A structure–activity relationship study on the antioxidant properties of 3-dithiocarbamic flavanones was performed. It was found that 6,8-dihalogenated flavanones exhibit better free radical scavenging than 6-monohalogenated flavanones. The influence of secondary amine moiety from the dithiocarbamic substituent has been also investigated, with *N*-pyrrolidinyl and *N*-morpholinyl carbodithioate derivatives exhibiting the highest antioxidant properties. Further investigation will target the influence of the substituents at the ring B on the antioxidant properties of dithiocarbamic flavanones provided by the hydrogen atom at the 2-position of the benzopyran scaffold. This is a preliminary report on the antioxidant properties of these 3-dithiocarmamic flavanoids and further studies will be reported soon.

## Figures and Tables

**Table 1:** Radical scavenging activities of compounds **5a**–**o** and **6** (IC_50_ values in nM) *.

Compound		DPPH	ABTS^+^	FRAP
(6-Bromo)	**5a**	277.47 ± 0.5	26.77 ± 0.2	89.73 ± 0.3
	**5b**	357.75 ± 0.7	26.14 ± 0.2	94.71 ± 0.3
	**5c**	257.11 ± 0.4	29.50 ± 0.3	84.72 ± 0.2
	**5d**	198.35 ± 0.4	28.02± 0.2	83.72 ± 0.4
	**5e**	240.93 ± 0.5	22.81 ± 0.1	77.85 ± 0.2
(6,8-Dibromo)	**5f**	165.67 ± 0.7	11.81 ± 0.1	12.46 ± 0.2
	**5g**	175.40 ± 0.8	14.20 ± 0.2	14.55 ± 0.3
	**5h**	119.11 ± 0.2	10.27 ± 0.2	10.01 ± 0.2
	**5i**	141.58 ± 0.4	16.78 ± 0.3	18.88 ± 0.4
	**5j**	120.08 ± 0.1	13.75 ± 0.3	14.54 ± 0.4
(6,8-Diiodo)	**5k**	166.73 ± 0.5	16.85 ± 0.1	37.45 ± 0.2
	**5l**	152.43 ± 0.4	14.77 ± 0.1	16.51 ± 0.1
	**5m**	206.05 ± 0.1	15.78 ± 0.2	47.54 ± 0.3
	**5n**	132.48 ± 0.2	16.70 ± 0.3	59.68 ± 0.3
	**5o**	127.02 ± 0.3	13.00 ± 0.2	15.16 ± 0.1
**6**		-	-	-
Ascorbic acid		74.17 ± 0.2	15.64 ± 0.1	27.18 ± 0.2
BHT		179.32 ± 0.5	14.66 ± 0.2	75.68 ± 0.3

* Mean values from triplicate tests. Data are shown as the mean ± standard deviation.

## Data Availability

Data is contained within the article or Supplementary Material.

## Supplementary Materials

The following supporting information can be downloaded at: https://www.mdpi.com/article/10.3390/antiox13080963/s1. Elemental analysis and copies of ^13^C-NMR spectra.
