# Supplementary material for: A Structure–Activity Relationship Study on the Antioxidant Properties of Dithiocarbamic Flavanones
Source: Antioxidants (Basel). 2024 Aug 8;13(8):963. doi: 10.3390/antiox13080963 (PMC11351990; doi:10.3390/antiox13080963)
Supplement: Supplementary file 1 [file antioxidants-13-00963-s001.zip › antioxidants-3113721-supplementary.pdf]

A structure-activity relationship study on the antioxidant properties of dithiocarbamic flavanones

M. Lucian Birsa and Laura G. Sarbu

Supplementary Material

1. Elemental analysis **S2**

2. Copies of ^{13}C NMR spectra **S3-S6**

1. Elemental analysis

Elemental analyses (C, H) were conducted using a CE440 Elemental Analyser; the results were found to be in good agreement (±0.3%) with the calculated values.

Table S1. Elemental analysis data for compounds **5h**–**j, 5m, 5n**.

Compound	% C		% H	
	calcd.	found	calcd.	found
**5h**	42.76	42.91	2.87	2.69
**5i**	43.81	44.05	3.15	3.29
**5j**	41.58	41.74	2.79	2.64
**5m**	36.63	36.84	2.46	2.59
**5n**	37.66	37.91	2.71	2.58

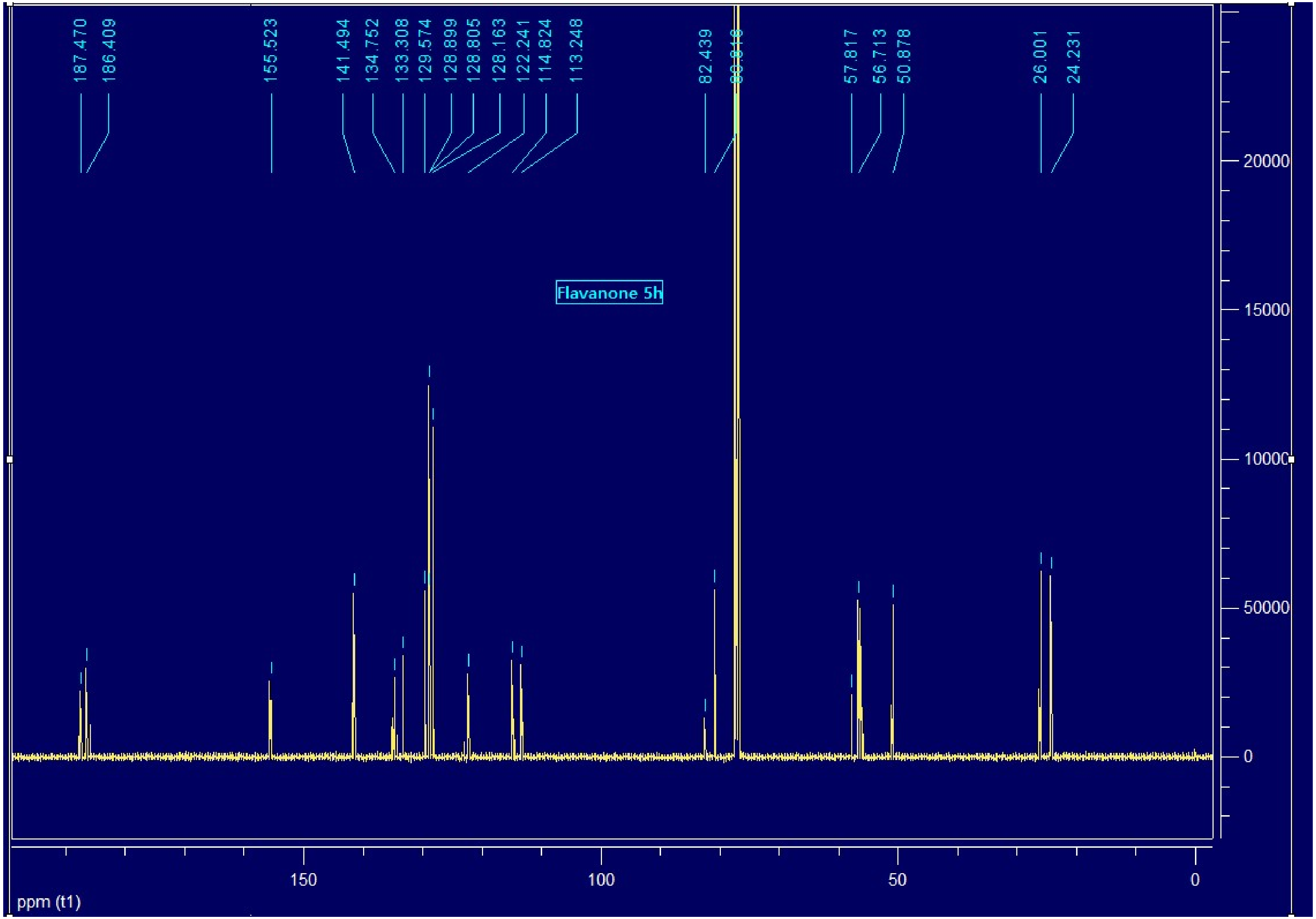
187.470
186.409
155.523
141.494
134.752
133.308
129.574
128.899
128.805
128.163
122.241
114.824
113.248
82.439
57.817
56.713
50.878
26.001
24.231
Flavanone 5h
20000
15000
10000
50000
0
150
100
50
0
ppm (t1)

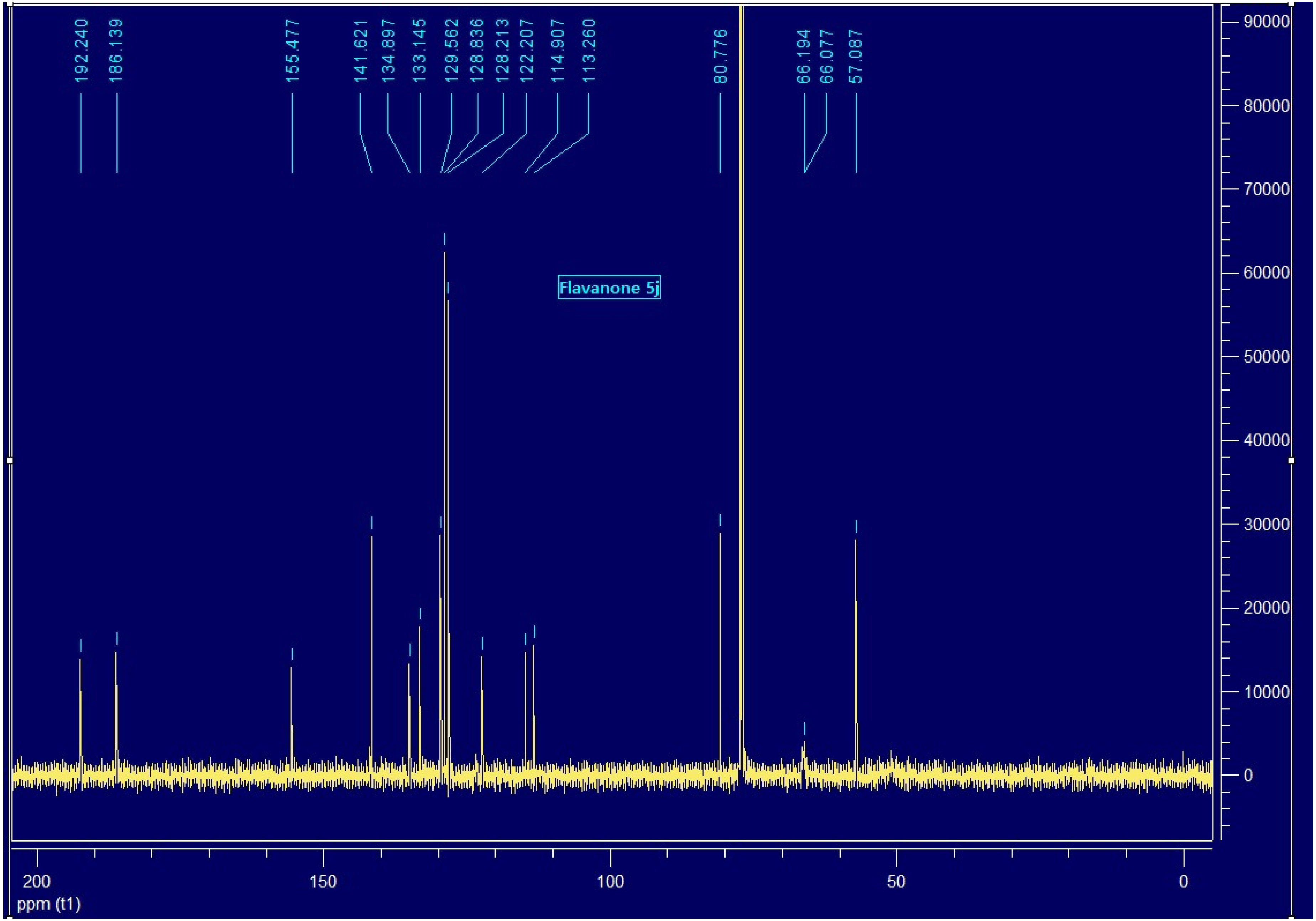
192.240
186.139
155.477
141.621
134.897
133.145
129.562
128.836
128.213
122.207
114.907
113.260
80.776
66.194
66.077
57.087
Flavanone 5j
90000
80000
70000
60000
50000
40000
30000
20000
10000
0
200
150
100
50
0
ppm (t1)

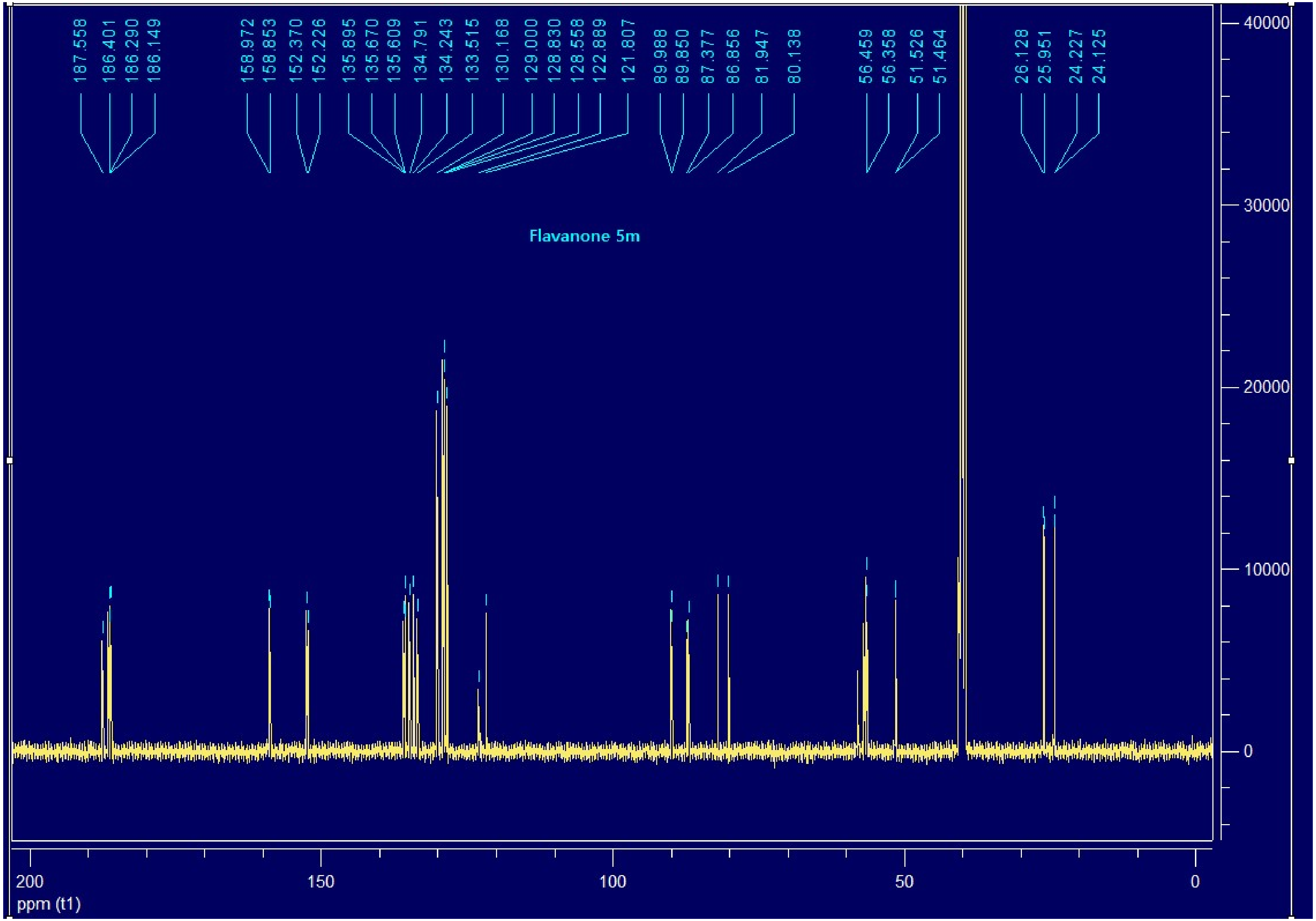

187.558
186.401
186.290
186.149
158.972
158.853
152.370
152.226
135.895
135.670
135.609
134.791
134.243
133.515
130.168
129.000
128.830
128.558
122.889
121.807
89.988
89.850
87.377
86.856
81.947
80.138
56.459
56.358
51.526
51.464
26.128
25.951
24.227
24.125
Flavanone 5m
40000
30000
20000
10000
0
200
150
100
50
0
ppm (t1)

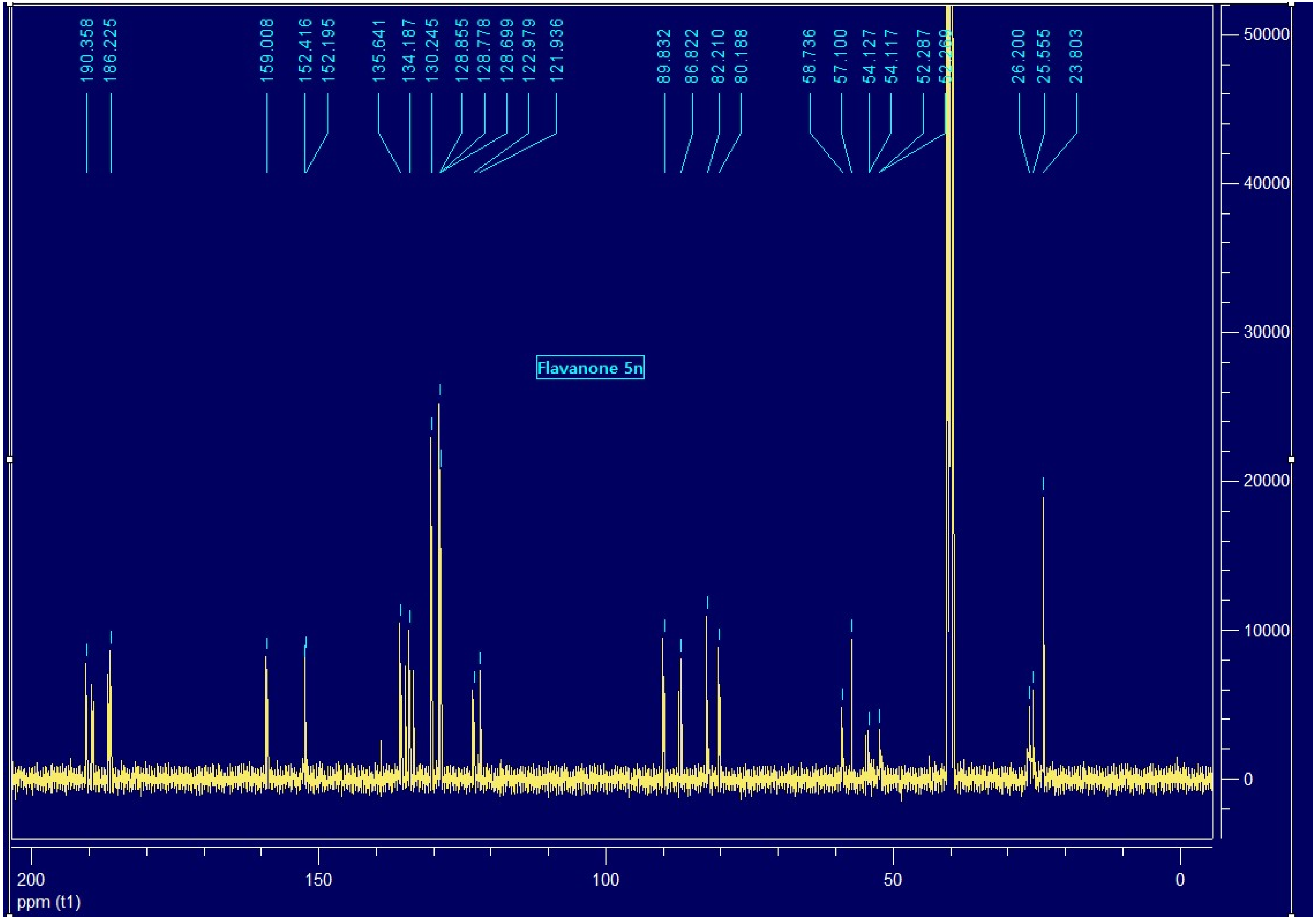
190.358
186.225
159.008
152.416
152.195
135.641
134.187
130.245
128.855
128.778
128.699
122.979
121.936
89.832
86.822
82.210
80.188
58.736
57.100
54.127
54.117
52.287
26.200
25.555
23.803
Flavanone 5n
50000
40000
30000
20000
10000
0
200
150
100
50
0
ppm (t1)
